# Sterile inflammation in liver transplantation

**DOI:** 10.3389/fmed.2023.1223224

**Published:** 2023-08-10

**Authors:** Riley Kahan, Paul L. Cray, Nader Abraham, Qimeng Gao, Matthew G. Hartwig, Justin J. Pollara, Andrew S. Barbas

**Affiliations:** Duke Ex-Vivo Organ Lab (DEVOL)—Division of Abdominal Transplant Surgery, Duke University, Durham, NC, United States

**Keywords:** machine perfusion, sterile inflammation, liver transplant, DAMPS (damage associated molecular pattern molecules), liver disease

## Abstract

Sterile inflammation is the immune response to damage-associated molecular patterns (DAMPs) released during cell death in the absence of foreign pathogens. In the setting of solid organ transplantation, ischemia-reperfusion injury results in mitochondria-mediated production of reactive oxygen and nitrogen species that are a major cause of uncontrolled cell death and release of various DAMPs from the graft tissue. When properly regulated, the immune response initiated by DAMP-sensing serves as means of damage control and is necessary for initiation of recovery pathways and re-establishment of homeostasis. In contrast, a dysregulated or overt sterile inflammatory response can inadvertently lead to further injury through recruitment of immune cells, innate immune cell activation, and sensitization of the adaptive immune system. In liver transplantation, sterile inflammation may manifest as early graft dysfunction, acute graft failure, or increased risk of immunosuppression-resistant rejection. Understanding the mechanisms of the development of sterile inflammation in the setting of liver transplantation is crucial for finding reliable biomarkers that predict graft function, and for development of therapeutic approaches to improve long-term transplant outcomes. Here, we discuss the recent advances that have been made to elucidate the early signs of sterile inflammation and extent of damage from it. We also discuss new therapeutics that may be effective in quelling the detrimental effects of sterile inflammation.

## Sterile inflammation: what is it?

Inflammation is the body’s coordinated response to injury or infection, serving to eradicate potential dangers, promote tissue regeneration, and create an environment supportive of an adaptive immune response. However, when immune cells are recruited to areas of cellular injury and death in the absence of microbial threats and initiate over-compensatory defense mechanisms, “sterile inflammation” occurs. This is caused by the immune detection of products released from damaged or dead cells and is currently understood as the body’s “damage-control” mechanism, attempting to contain uncontrolled cell death and stimulate tissue repair for the return to homeostasis (1).

Sterile inflammation is a significant factor in the progression of liver disease and a determinant of outcomes after liver transplantation. The liver is responsible for detoxification and metabolic processing, and its physical connection with the gastrointestinal tract allows for first-pass screening and metabolism of blood products. Constant exposure to toxins can lead to prolonged inflammation and damage, progressing into various stages of disease that may ultimately require transplant for treatment.

**Figure 1:**
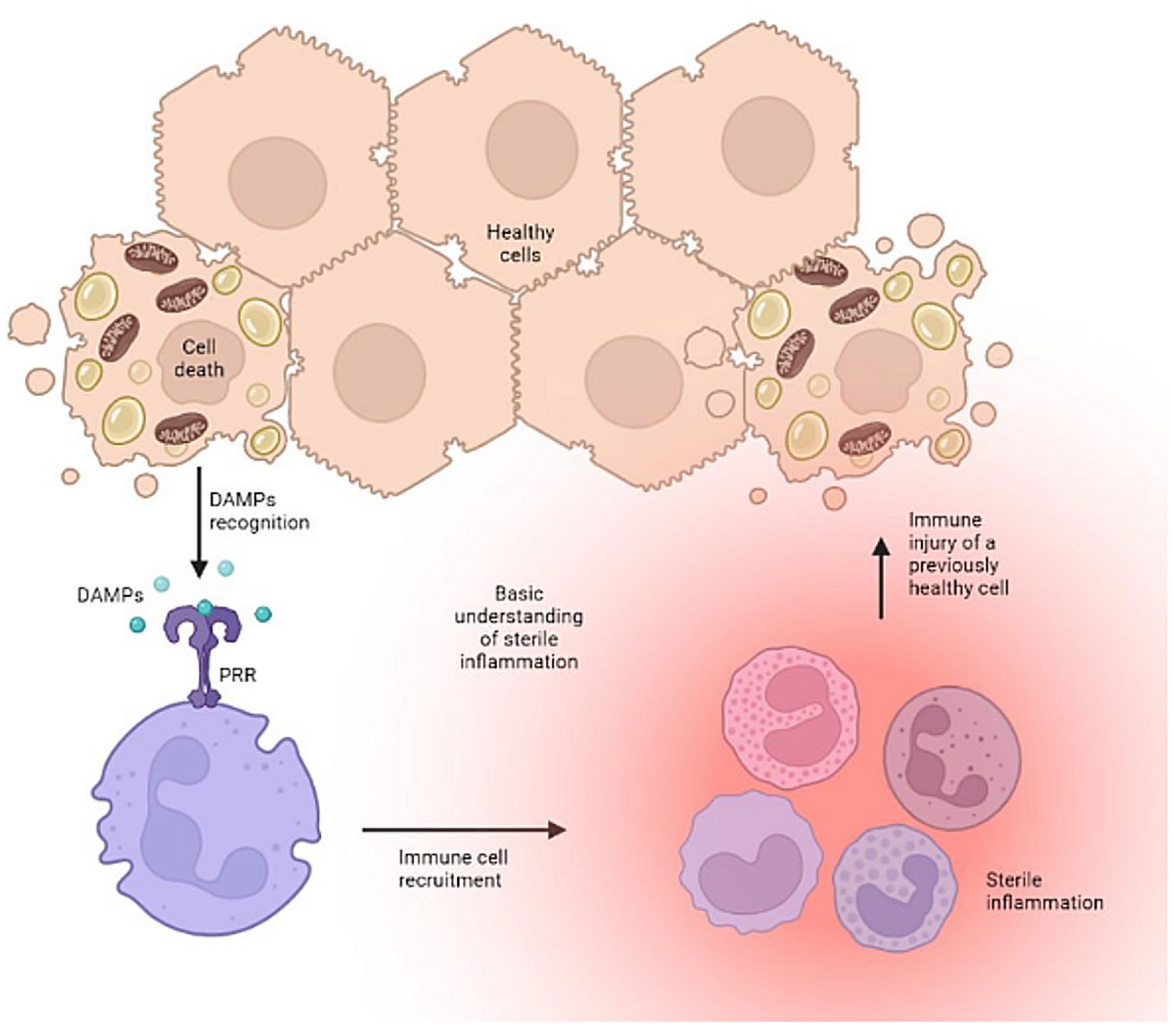
The vicious cycle of uncontrolled sterile inflammation. Created with biorender.com.

The development of pathogen-free liver disease is well characterized (2, 3). Disease development stems from shared signaling pathways for both repair and defense, which when imbalanced, can lead to excessive activation of the innate immune system causing self-injury. Notably, the body’s injurious effort to defend and repair tissue stems from the innate immune system’s overt response to certain conserved molecular motifs, or damage associated molecular patterns (DAMPs). This mechanism produces the secondary signals necessary to activate an aggressive adaptive immune response and cause dysfunction and/or disease. It serves as a bridge between the innate and adaptive immune responses, and in the liver, initiates a cyclic pattern of immune cell recruitment, parenchymal cell death, and further DAMP release (Figure 1).

Organ procurement, storage, and reimplantation all provide contexts for sterile inflammation to occur (4). When an organ is transplanted, it undergoes two types of injury: ischemic injury during procurement and storage, and reperfusion injury during reimplantation (5). Ischemia and reperfusion injuries (IRI) can cause cell death and trigger sterile inflammation that primes the adaptive immune system, which ultimately contributes to antibody and T cell mediated rejection of the transplanted liver (6, 7). Identification of specific biomarkers has allowed for a closer look at the inflammatory and immunological state of an organ both before and after transplant (8). In this review, we discuss some of the key cellular and molecular cues of sterile inflammation, the immunological implications of the organ recipient disease states, the effect of sterile inflammation on the donor organ, and finally, recent advances in machine perfusion and attempts to expand the liver donor pool.

## DAMPS and biomarkers

Many DAMPs have been associated with sterile inflammation, and indeed it has been shown that some DAMPs are not only indicators, but also activators. This distinction is important for understanding whether a DAMP can modulate an immune response, trigger one, or is largely evidence that sterile inflammation is/did occur. Here, we describe common DAMPs, and evidence to support their role as activators, immunomodulators, or indicators of an inflammatory response.

### High mobility group box 1

High mobility group box 1 (HMGB1), a nuclear gene regulatory protein, is principally found residing in the nucleus, but can be secreted in response to cell stress or injury. Secreted HMGB1 interacts with many toll-like receptors (TLRs) and receptor for advanced glycolytic end-products (RAGE) to act as a proinflammatory alarmin DAMP (9–12) and activate innate immune responses (13, 14).

**Figure 2:**
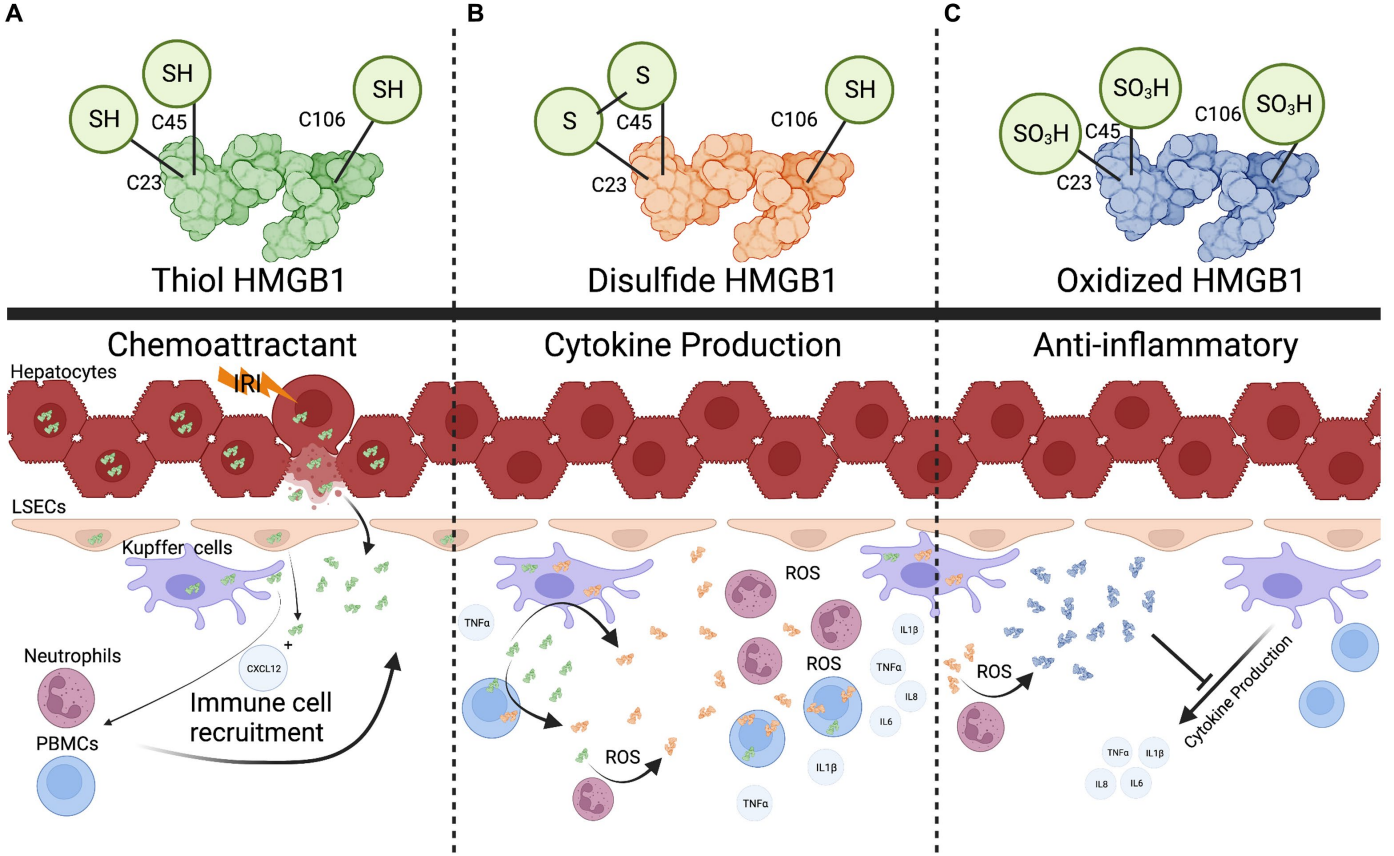
The multifaceted role of HMGB1. **(A)**, the fully reduced form of HMGB1 can form heterocomplexes with other proteins such as CXCL12 to act as a chemokine to recruit immune cells. **(B)**, Recruited immune cells can uptake reduced HMGB1 and oxidize it into a pro-cytokine stimulating (disulfide) form. Reactive oxygen species released in the extracellular space can also convert HMGB1. **(C)**, Once a threshold amount of ROS is produced, the disulfide form is converted into a completed oxidized form that can potentially dampen cytokine production. Created with biorender.com.

An important aspect of sensing HMGB1 as a DAMP is that changes to the redox state of HMGB1 are often required for proinflammatory activation (15) (Figure 2). This has been demonstrated in several settings, including liver transplantation (16–21). For example, it has been shown that the thiol form of HMGB1 can form heterocomplexes with CXCL12 and act as a potent chemokine, drawing immune cells to the site of injury (Figure 2A) (22, 23). In contrast, cytokine production in response to HMGB1 sensing has been linked to a different HMGB1 redox state. Sosa et al. (19) found that while portal blood following allograft reperfusion had elevated levels of HMGB1, only patients with significant ischemia-reperfusion injury (IRI) had increased levels of disulfide-HMGB1 as well as increased TLR4 mediated production of TNFα. Further, the investigators demonstrated that disulfide-HMGB1 causes a positive feedback loop for HMGB1 secretion, wherein uptake of disulfide-HMGB1 by monocytes leads to the cells translocating and secreting their own HMGB1 (Figure 2B) (19). Liver flush effluent from transplant patients with subsequent histopathological IRI activated immune cells *ex vivo* when compared to cells exposed to flush effluent from injury free controls (24). This suggests that changes to the redox state are needed for HMGB1 to be a triggering DAMP in the setting of IRI. In other circumstances redox forms of HMGB1 are not required for pro-inflammatory signaling. Monocytes and innate immune cells have been shown to take up HMGB1 as well as secrete different forms of HMGB1, altering the function of HMGB1 in the local inflamed space (25, 26). Finally, the completely oxidized form of HMGB1 has been suggested to assist with the injury resolution and promote an anti-inflammatory state (Figure 2C) (27). Using a MHC-II mismatch rat orthotopic liver transplant model, Chen et al. (28) show that HGMB1 activated dendritic cells, which in turn led to differentiation of CD4^+^ T cells into Th1 and Th17 cells and ultimately promoted acute liver rejection. Administration of the HMGB1 inhibitor glycyrrhizic acid extended graft survival time and improved graft function (28). As a biomarker in human transplant, HMGB1 has also been shown to be a potential predictor of primary non-function and early allograft dysfunction (29–31). However, it is still unclear what the redox status of HMGB1 was in these studies. Recent work in the setting of machine perfusion has shown that HMGB1 levels correlate with levels of proinflammatory cytokines in the perfusate, and suggest that removal or inhibition of HMGB1 before implantation could improve graft outcomes (30).

Additionally, HMGB1 has been shown to be an important marker for biliary atresia, and to also have a likely pathogenic role in this setting as anti-HMGB1 treatment increased survival in a murine biliary atresia model (32). This could translate into the post-transplant space where biliary complications are common after liver transplant and may be a marker for biliary health (33). Recent advancements have begun to clarify the diverse roles of HMGB1which are tuned by redox state. HMGB1 starts as a proinflammatory molecule for immune cell recruitment, then converts into a cytokine production stimulating molecule once a threshold of cells has been recruited, and finally into a tolerogenic form to help assist with repair once a sufficient amount of reactive oxygen species (ROS) has been produced (Figure 2) (16, 27). With these recent advancements, more work needs to be done to define HMGB1’s redox status when measured as a DAMP, and identify how this can be manipulated to minimize the contribution of HMGB1 inflammatory state in the setting of liver transplantation.

### Heme and heme-oxygenase 1 (HO-1)

Heme is an essential component of hemoglobin, allowing for oxygen transport and storage in the blood. However, when dissociated from apo-heme proteins that allow it to function in beneficial ways, it becomes free-heme, an iron containing porphyrin that is capable of causing cell damage and tissue injury due to its ability to catalyze formation of reactive oxygen species (34). Relatedly, heme-oxygenase 1 (HO-1) is a protein that functions to lower free-heme concentrations and prevent heme-mediated oxidative injury (35).

Free heme is a potentiator of inflammation that requires a trigger signal to produce an effect (36, 37). Heme activates the NLRP3 inflammasome through Syk signaling which is mediated by the generation of reactive oxygen species (ROS) (36, 38, 39). Additionally, it has been shown that heme can cause complement activation and accumulation on sinusoidal endothelial cells via a P-selectin mediated pathway (40, 41). Heme also activates TLR4, which exacerbates the accumulation of complement on the liver endothelial surface (41). However, in an isolated *in vitro* system, high levels of heme were unable to induce a strong TLR4 response, corroborating the theory that an additional signal is needed (37). This is also seen in a mouse model of free heme from sickle cell disease, where P-selectin activation of TLR4 is required as a secondary signal in heme-induced inflammation (42).

Conversely, the oxidation state of the iron in hemin, the oxidized form of heme, plays a pivotal role in the ability of heme to activate HO-1 and attenuate NLRP3 inflammasome activation and cellular IL-1β production (39, 43). Macrophages are one of the main responders to heme, and clodronate liposome mediated depletion of macrophages significantly improves survival of severe hemolysis (36, 44). Recently, it has been shown that HO-1 in donor myeloid cells is pivotal in controlling Graft vs. Host Disease (GVHD) in recipients of hematopoietic stem cell transplantation (45). Briefly, Spilleboudt et al. (45) show that donors with mutations in *HMOX1* were associated with lower HO-1 expression and were more likely to develop severe GVHD compared to those that had higher expression HO-1. Mechanistic exploration of HO-1 by Nakamura et al. (46) shows that HO-1 upregulates SIRT1 which increases p19 levels and subsequently sustains p53 activity in repressing macrophage activation. Their work corroborates that lower levels of HO-1 lead to exacerbated pro-inflammatory responses after liver IRI.

Cellular systems are not isolated, and stresses in one compartment can lead to additional stress in another. Indeed, oxidative stress and endoplasmic reticulum (ER) stress are strongly interconnected (47). As specialized macrophages, Kupffer cells are one of the main constitutive expressors of HO-1 in the liver and recent work has suggested that regulation of ER stress may be one method of protecting against IRI (48). Cai et al. (49) show that loss of IRE1a, one of the main transducers of ER stress, is protective against IRI. Taken together, this collective body of work shows that regulation of iron, by controlling heme levels, is required for maintaining homeostasis, and modulation of the heme-HO-1 axis is a viable therapeutic target for IRI and liver transplant. In fact, there are already clinical trials for HO-1 modulation via heme arginate in deceased donor kidney transplant (50).

For further reading, a more in-depth look at HO-1 in liver transplant IRI was recently reviewed (51) as well as for other Hemoglobin derived DAMPs (52).

### ATP

While ATP is a requirement for maintaining cellular homeostasis, extracellular ATP is a potent pro-inflammatory DAMP in IRI and transplant (53–55). Extracellular ATP is released during programmed cell death, such as apoptosis and necroptosis, but it is also released in a regulated fashion via the Pannexin channels (56, 57). Recognition and control of extracellular ATP is tightly controlled by numerous cells in the inflamed space. In addition to the release of ATP by damaged cells, local activated macrophages release ATP to stimulate other innate immune cells (53). Here, cellular response is context driven, in that cells expressing large amounts of P2X will be driven into a pro-inflammatory state, whereas P2Y and P1 will shift the cell into an anti-inflammatory phenotype (58). As immune cells are recruited to the site of inflammation, regulatory T and B cells dampen the ATP signal by converting ATP into its less energetic forms ADP and AMP via CD39 (59, 60) and finally into adenosine by CD73, which as previously mentioned, has been demonstrated to be a potent immunosuppressant (61, 62). Adenosine is taken up very rapidly via endothelial cells and has a relatively short half-life of about 7 s in blood (63). This inhibition by adenosine is particularly important in controlling T cell and Natural Killer (NK) cell responses in the local space and proper regulation of the ATP:adenosine axis is required for attenuating IRI and immune mediated rejection *in vivo* (64).

In the context of liver transplant, modulation of the ATP:Adenosine axis has only recently been assessed. Kelly et al. (65) showed that in a small-for-size porcine liver transplant model, adenosine administration greatly improves survival due to increases in hepatic artery flow. Without this increase, vasoconstriction occurs and effectively strangles the small graft via vasospasm causing necrosis, graft loss, and ultimately death (65). This finding translated well to the clinic, with Zhu et al. (63) showing similar increases in hepatic artery flow when patients were administered adenosine over a 30 min period following liver transplant. Similarly, Czignay et al. (66) recently showed that activation of the adenosine A2A receptor in a porcine model of DCD liver transplantation improved graft function, tissue microcirculation, and overall survival.

*In vivo* work by Pommey et al. (67) show, in an extended static cold storage model of syngeneic liver transplant, that overexpression of CD39 in the donor organ was protective, reflected by liver function tests and histological scoring, due to reduced number of resident CD4^+^ T cells. They then go on to show that this protection is lost when transgenic donors had their bone marrow replaced with wild type bone marrow prior to the liver transplant. Analysis of the thymus of these CD39 overexpressing mice showed T cell lymphopenia and a disruption of T cell maturation (67). This is corroborated by Yoshida et al. which shows that CD39 dependent ATP regulation plays a major role in attenuating IRI and immune mediated rejection *in-vivo* (64). Despite these interesting results, Baroja-Mazo et al. (68) suggest that adenosine may not be a viable option for improving long-term tolerance after liver transplant. Since regulatory T cells generate large amounts of adenosine from ATP using CD39, it was hypothesized that addition of exogenous adenosine would be beneficial for promoting tolerance in an immunosuppression withdrawal trial. In contrast, *in vitro* studies with isolated human T cells demonstrated that high amounts of adenosine inhibit the function of both T effector cells and regulatory T cells, thus limiting potential for a tolerogenic benefit. Together, this suggests that the ATP:CD39:Adenosine pathway specifically in lymphocytes may be a viable control mechanism for tuning the immune response.

### Mitochondrial DNA

Mitochondria, as one of the main controllers of cell fate, have been implicated as a major biomarker for severity of inflammatory disease (69, 70). In a seminal paper, Zhang et al. (70) demonstrated that mitochondrial derived DAMPs elicit PMN activation and mobilization. Of these DAMPs, they describe mitochondrial DNA (mtDNA) as being a key pro-inflammatory molecule. However, they show that only co-stimulation of mtDNA with n-formyl peptide, a potent PMN stimulator, was able to elicit pro-inflammatory cytokine production when compared to control. Investigation into the inflammatory properties of mtDNA by Collins et al. (71) show that oxidation of the mtDNA is paramount for eliciting the immune response, and mtDNA with no oxidized lesions fails to elicit an immune response. Hamilton et al. (72) show that there are only one to two lesions of oxidized 8-oxo-2-deoxyguanosine per 100 mitochondrial genomes in the healthy mouse. This suggests that the oxidation state of the mtDNA is a key characteristic that influences its contribution as an inflammatory mediator. Further, mtDNA can be a major component of neutrophil extracellular traps (NETs) (73, 74). This reinforces the idea that mtDNA is a biomarker that indicates a large scale pro-inflammatory event has occurred, rather than mtDNA being the primary activator of inflammation.

In the transplant space, mtDNA have been shown to be correlated with primary graft dysfunction in the lung, and our group has shown that mtDNA is a biomarker of early allograft dysfunction for both liver and kidney transplant (75–77). In trauma patients, not all cell-free mitochondrial DNA is membrane free and is bound in complexes from sizes 0.45 μm to 5 μm, which roughly corresponds to the size of mitochondria from different cell types (78, 79). Our previous work corroborates this by showing that a majority of the mtDNA released is still bound in extracellular mitochondria (75). This compartmentalization could indicate that the mtDNA is indicative of dysfunctional mitochondria and that is contributing to the inflammatory state, rather than the mtDNA alone. Regardless, more work needs to be done to understand what role mtDNA has in liver transplant.

### Interleukin 33

Interleukin 33 (IL-33) is a nuclear protein that acts as an alarmin and DAMP when released from cells, and has recently been shown to have clinical relevance in acute liver tissue injury (80). Circulating levels of IL-33 were found to increase shortly after reperfusion in the context of liver transplant (81), and serum concentrations of IL-33 correlated with increased liver IRI (82). Mechanistic studies performed in mouse models of liver IRI demonstrated that IL-33 exacerbated sterile inflammation in the liver by recruiting neutrophils (81) in an ST2 receptor dependent manner (81). Interestingly, however, Ferhat et al. (83) review IL-33 and cite previous studies that have found pretreatment with IL-33 before IRI injury to instead be protective, via interactions with T cells to induce an anti-inflammatory state. Because of this, IL-33 remains a captivating and understudied DAMP that is capable of predicting liver transplant outcomes as well as mediating the innate and adaptive immune system to protect from, or exacerbate, sterile inflammation after reperfusion.

## Clinical relevance of sterile inflammation in liver disease and transplant

While the etiology and mechanisms of sterile inflammation have been studied extensively in animal models, it still poses a significant challenge for transplant outcomes in the clinical setting. Sterile inflammation remains a key contributor to the progression of liver diseases and poisoning that are the most common causes of end stage organ failure necessitating transplant: alcoholic and non-alcoholic steatohepatitis, and acetaminophen intoxication.

### Alcoholic steatohepatitis

Sterile inflammation is the key underlying mechanism behind alcoholic steatohepatitis (ASH) (84). ASH is characterized by fatty accumulation in the liver accompanied by inflammation after chronic alcohol consumption. In early stages of ASH, ethanol induced hepatocyte insult causes apoptosis and initiates macrophage-mediated inflammation. Chronic alcohol consumption causes persistence of toxic acetaldehyde production and oxidative stress after cytochrome P450 2E1 (CYP2E1) mediated ethanol metabolism in hepatocytes (85). In late-stage ASH, when hepatocytes begin dying via receptor interacting serine/threonine kinase (RIPK1) mediated necroptosis, the DAMPs released by the lytic nature of this specific cell death process cause neutrophilic, macrophagic, and/or inflammasome mediated inflammation to ensue (84). As previously mentioned, DAMPs such as HMGB1, DNA, ATP, adenosine, and fibrinogen are among the molecules recognized by TLRs on immune cells that can trigger this inflammatory response (86, 87).

The innate and adaptive immune systems are both heavily involved in ethanol induced hepatocyte insult and successive DAMP release: Kupffer cells respond to DAMPs by producing pro-inflammatory cytokines such as TNFα and IL-1β (88). Infiltrating peripheral monocytes then respond by differentiating into M1-like hepatocytic macrophages via Notch-1 dependent reprogramming to increase the magnitude of cytokine production via increase of pro-inflammatory molecules such as IL-12, IL-18, and IL-23 (89). Subsequently, CD4^+^ helper T-cells infiltrate the liver and initiate events of mass cytokine and chemokine release in order to further activate macrophages and recruit CD8^+^ cytotoxic T cells (90, 91), which ultimately act to cause severe inflammation, tissue fibrosis, and death of functional hepatocytes (92, 93). Importantly, this injurious cascade is significantly mediated by the interaction between DAMPs and toll like receptors (TLRs) on innate immune cells (84).

Once manifested, ASH acts as a precursor to cirrhosis, hepatitis, or cancer, and end stage disease often requires liver transplantation as a form of long-term management as the functional volume of the liver decreases (94). Management of liver allografts post-transplant is often complicated by immune system sensitization. Specifically Purohit et al. (95), describes an increased permeability of the intestine to endotoxins in response to chronic alcohol consumption, resulting in further inflammatory changes in the liver. In addition, alcohol and its metabolites stimulate the production of large quantities of NF-kB and stimulate TLR’s to mediate the synthesis and release of pro-inflammatory cytokines and factors, impairing systemic immune regulatory function (95).

### Non-alcoholic steatohepatitis

Non-alcoholic steatohepatitis (NASH), the second leading indication for liver transplantation in the United States, is not as well understood as ASH (96). A variety of agents including high dietary fat and sugar consumption, impairment of fatty acid disposal (metabolic dysfunction), diabetes, obesity, or genetics can all lead to lipotoxic overload in the liver (97). The downstream effects of this overload include excessive stress on endoplasmic reticulum, mitochondrial dysfunction, hepatocellular injury, inflammation, and undesirable apoptosis, all of which are stimuli for fibrosis and malignant transformation in the liver (97).

Like ASH, chronic inflammation observed in NASH is driven by a family of proteins referred to as CIDE proteins that regulate lipid homeostasis by facilitating the accumulation of chained fatty acids in hepatocytes in order to maintain lipid equilibrium in circulation. When this protective mechanism is overwhelmed, stressed hepatocytes undergo apoptosis via increased expression of CIDE proteins (98). This readily causes DAMP (HMGB1, DNA, ATP) release and initiates an injurious cascade similar to that observed in ASH (84). Excess circulation of fatty acids and cholesterol in the blood can also directly activate Kupffer cells to recruit neutrophils, monocytes, and NK cells to the liver, exacerbating the inflammatory response. This causes further release of cytokines and chemokines that subsequently result in an accumulation of Th17 CD4^+^ Helper T Cells in the liver, a hallmark of NASH. Positive feedback interplay between hepatocyte cell death and inflammatory activity ensues (99).

Due to high levels of circulating DAMPs, patients with NASH also present with significantly increased levels of C3, activating multiple complement cascades and associated adaptive immune pathways upon DAMP recognition (100). In terms of stimulating the adaptive immune system, these complement proteins stimulate B cells to aggregate, produce pro-inflammatory mediators. and present antigens to CD8 T cells via MHC class I proteins (101–103).

Once NASH advances to fibrotic cirrhosis the only treatment is liver transplantation. However, patients with metabolic syndrome that develop NASH severe enough to necessitate liver transplantation often develop de-novo NASH in the new liver (104). This is caused by persisting metabolic syndrome in these patients; liver transplantation treats their life-threatening symptoms, but does not reverse the etiology of the disease (105). The immune systems of these patients are still primed for chronic hepatic inflammation and buildup of fibrotic tissue in the implanted liver (106).

### Acetaminophen intoxication

In contrast to the chronic diseases where end stage liver failure is well defined, more abrupt transplant needs present after acute toxicity and subsequent liver failure. Intoxication from acetaminophen overdose is the most frequent cause of acute liver failure in the United States (107) and contributes to 70,000 hospitalizations each year (108). Acetaminophen intoxication occurs when enough is consumed to overwhelm the few mechanisms of metabolism and clearance that occur in the liver. Upon initial exposure, the majority of acetaminophen is conjugated with glucuronic acid or sulfate and is subsequently excreted via nephrons in the kidney (109). If this mechanism becomes saturated, cytochrome P450 enzymes in the liver begin to metabolize acetaminophen by converting it to another metabolite, *N-acetyl*-*p-*benzoquinone imine (NAPQI) (110). If acetaminophen is consumed at the therapeutic dose, the small amount of NAPQI that is produced can be quickly conjugated by the ample glutathione stores that exist in the liver. This conjugated form of NAPQI is then safely excreted in the bile. However, in the case of overdose, the glutathione stores of the liver are depleted in the presence of excess NAPQI. When this occurs, the un-conjugated NAPQI is plentiful and available to react with protein sulfhydryl groups to form acetaminophen protein adducts (109). These protein adducts become harmful when they interact with mitochondrial proteins and create dysfunctional forms of other critical proteins such as ATP synthase, HMG CoA synthase, and glutathione peroxidase. Dysfunctional mitochondrial activity caused by acetaminophen protein adducts is robustly characterized by the formation of harmful ROS and free radical compounds (111, 112). *In vitro* studies of acetaminophen intoxication in mouse hepatocyte cell lines have revealed acetaminophen protein adducts affect complex II of the electron transport chain, and are a major source of superoxides (113, 114). Once ROS are produced at a high enough concentration, a threshold is exceeded that will send cells into abortive necroptosis in an attempt to prevent further production of ROS (115). However, this inevitably leads to the release of DAMPs and positive feedback loop of sterile inflammation, mediated by crosstalk between the innate and adaptive immune systems. Our understanding of hepatic sterile inflammation and its underlying mechanisms is still evolving, and future research is required to further elucidate the relationship between liver disease and immune modulation in the clinical setting.

## Acute liver failure

As previously mentioned, acute liver failure (ALF) from drug toxicity presents as the most common source of liver failure in the United States. However, while acetaminophen is the most common agent, idiosyncratic responses to other drugs leading to acute liver failure have been reported (116). These responses provide a particular challenge to clinicians as the presentations are ubiquitous to liver damage in general (117–119). Liver enzyme testing (e.g., AST and ALT) provides a sensitive means of delineating liver damage, but is not very specific (117, 120). To further complicate the origin of acute liver failure, there is a genetic component to the sensitivity to drug induced hepatotoxicity (121).

While extensive work has been done describing the association of liver injury and DAMPs, there is still a need for a set of biomarkers that can help identify the mechanism of specific acute liver failure and distinguish failure from injury (117, 122). Roth et al. (123) show that IL-33 was upregulated in patients with end stage liver disease as well as patients with acute liver failure and chronic liver failure compared to healthy controls. While there was no difference in the amount of IL-33 in the injured cohorts, the amount of soluble ST2 (sST2), the decoy receptor for IL-33, was significantly different between the acute groups and the chronic only group. As previously described, the IL-33/sST2 axis is a promising area of study as elevated levels of sST2 have been implicated as a biomarker of worsening/negative outcomes in other types of injuries (124). In particular, sST2 correlated strongly with mortality within 30 days after acute myocardial infarction (125) and with acute cardiac allograft rejection (126). sST2 has also shown to correlate with the extent of disease and mortality associated with hepatitis B virus in acute on chronic liver failure (127). Together, these findings suggest that sST2 may be a viable biomarker for delineating liver injury and liver failure, but more work still needs to be done to understand why the level of sST2 is sustained and how this impacts subsequent immune processes.

## Autoimmune hepatitis

Autoimmune hepatitis (AIH) presents another challenge due to its cryptic pathogenesis (128). It has been shown to be caused by viral infection, exposure to certain drugs, and other insults (128). While there are effective treatments such as steroids, the disease can cause both acute and chronic injury and may progress to liver failure requiring transplant (128, 129). AIH occurs when individuals develop either autoantibodies or T cells against components of the liver after some triggering event (130). It is classically diagnosed by the presence of autoantibodies and elevated liver enzyme levels, but there have been instances of seronegative autoimmune hepatitis (131). Confirmation is normally done by liver biopsy, which comes with its own challenges for collection and analysis and drives the need for a less invasive diagnostic test and a better understanding of the different initiating pathways.

Given the diverse range of initiating insults that can start AIH, there is a challenge for developing an animal model that can be used to help describe the pathogenesis mechanistically (132). A T cell mediated development of AIH in mice has been described with the use of concanavalin A (133–135). This model is advantageous as it is easily reproduced with a single injection; however it only describes one possible mechanism of developing AIH (134).

In contrast to developing an animal model, some progress has been made showing that some DAMPs correlate with disease severity and treatment response in AIH (136–138). AIH patients had higher levels of circulating EN-RAGE (extracellular newly binding RAGE ligand) and lower sRAGE (soluble RAGE) compared to healthy controls (138). The RAGE pathway has been implicated in the expansion of the myofibroblast population (139) and activation of the monocyte/macrophage populations by EN-RAGE (140). The amount of sRAGE could be used to separate non-cirrhotic livers from cirrhotic livers, suggesting that the dampening capacity of the decoy receptor plays a major role in the development of cirrhosis (138). While analysis of the RAGE system may prove to be beneficial for delineating AIH from other conditions, more work needs to be done to understand which DAMPs are specific to AIH.

## Adaptive immunity after sterile inflammation; recipient pre-conditioning

As previously mentioned, liver transplantation is a life-saving procedure for patients with end-stage liver disease. For many years, the focus of research in this field has been on optimizing donor organs to improve outcomes. However, it is becoming increasingly clear that the immune system of transplant recipients requires further attention, as its activation and conditioning after pathology involving sterile inflammation likely plays a significant role in determining the balance between allograft tolerance and immunity upon implantation, ultimately determining the success of the transplant (141–143).

Determinants for outcomes in liver transplant recipients has largely been shifted away from HLA mismatching (144, 145) or the presence/absence of donor specific antibody (146). This is attributed to the liver’s ability to regenerate quickly, the capacity of Kupffer cells to remove activated complement and immune complexes, and the liver’s low expression of HLA class II antigens (147). On the other hand, the state of recipient immune systems has been heavily implicated in graft failure; underlying liver inflammation and increased secretion of cytokines after viral hepatitis, alcoholic and non-alcoholic steatohepatitis, and hepatic carcinoma may result in recipient immune preconditioning that increases the likelihood of graft failure after implant. As previously discussed, alcoholic steatohepatitis causes stress related hepatocyte apoptosis and subsequent DAMP release (148). HMGB1 release from hypoxic mitochondria is a hallmark of IRI injury in the liver (149). It is also well known that HMGB1 induces cytokine production and chemotaxis as its concentration increases in circulation (150). Other pathology, such as hepatitis B infection, can cause significant DAMP release and high concentrations of pro-inflammatory cytokines to exist in circulation (151). Logically, it is possible that liver transplant recipients who have increased circulating concentrations of DAMPs due to their transplant indicating condition are more likely to have higher incidence of poor outcomes after transplant due to pre-sensitized immune related complications.

Fortunately, there exist mechanisms that may provide opportunity for recipient immune conditioning. For example, Ono et al. (152) show that graft-infiltrating recipient CD11c^+^ dendritic cells are capable of recognizing donor MHC-I antigens and presenting them as “self” while expressing high levels of PD-L1, disarming the T effector cell proliferative response. In a murine model, Khanna et al. (153) show that treating liver transplant recipients with liver-derived myeloid dendritic cells induces native T cells to produce anti-inflammatory IL-10. Additionally, it has been shown that in response to continuous, low level LPS stimulation, Kupffer cells will begin to secrete anti-inflammatory IL-10 and TGF-b (154, 155). This phenomenon, termed “LPS tolerance,” could potentially be used to pretreat liver transplant recipients. Similarly, liver sinusoidal endothelial cells have also been shown to utilize Fas–Fas ligand and CD80/86 to cause immune tolerance inducing effects on both naive CD4^+^ and CD8^+^ T cells (156, 157); these mechanisms could also be potential targets for recipient immune preconditioning.

Previously much of the research focused on improving outcomes in liver transplant recipients is centered around donor optimization, attempting to answer questions like: how can we assess, treat, and ultimately preserve the viability of a donor organ? However, it is clear that further research must address the immunologic state of the recipient’s innate *and* adaptive immune systems. It is likely that the immune activation and conditioning, or lack thereof, of the recipient has significant influence on the post-transplant viability of liver allografts. Considering the possibility of pre-transplant adaptive immune preconditioning in transplant recipients presents a gap in the current literature that may explain current unanswered questions regarding improving outcomes in liver transplant recipients. Nevertheless, exploring techniques to improve the quality and alter characteristics of donor organs is still critical in improving transplant outcomes.

## Therapeutic interventions

### Pharmacologic

The ability of pharmacological therapies to abrogate hepatic sterile inflammation has historically relied on antioxidants (to reduce quantity of reactive oxygen species), vasodilators, and anesthetics, all of which yield inefficient and insufficient results (158). Because of this, approaches to mitigating sterile inflammation after liver transplantation have changed from retroactive to proactive. Instead of attempting to interrupt sterile inflammation once it has begun, the paradigm for intervention has shifted to approaches that halt inflammatory pathways before they can occur. There exist a variety of potential therapies that target the intermediates between DAMP production/recognition and proliferation of pro-inflammatory signals and effectors. Two cytokines that have previously attracted attention in this context are IL-23 and IL-17A, which are believed to cooperatively act to induce mass neutrophil infiltration in hepatic IRI (159). These cytokines, released by Kupffer cells and hepatic NK cells, respectively, recruit neutrophils in a manner accessory to the TLR-4/NF-κB/HMGB1 pathway, and may prove to be an effective target for inhibition to downregulate IRI. Similarly, Gao et al. (84) found that intravenous injections of melatonin may have a mitigation effect on hepatic IRI after partial liver ischemic injury in a manner dependent on inhibition of the NF-KB pathway. Since they found that NF-kB levels were increased after IRI but decreased with melatonin treatment, this reinforces the potential of targeting the NF-kB pathway to prevent sterile inflammation.

Another class of potential therapeutics are senolytics, which have recently become of interest for optimizing older organs for transplantation in the context of inflammation (160). Recent evidence has supported the idea that age is a strong predictor of organ susceptibility to IRI in the setting of liver transplantation (161). The reasoning for this is postulated to revolve around the reduced capacity of mitochondria in aged livers to produce ATP and thus retain intracellular energy after IRI (162). This effect has been observed in humans (163) and investigated in translational experiments using rat liver IRI models (164). Conveniently, senolytics act to selectively deplete senescent cells (165), theoretically conditioning older organs to diminish age-related inflammation that would inherently cause an organ to be less desirable for transplant (166). These therapeutics have this effect by acting upon many pathways that have been previously studied, such as the PI3K/AKT pathway (167). Although this is an attractive approach these drugs are still in very preliminary stages of study, and the effects of which will need to be further interrogated.

Additional recently investigated mechanistic targets and therapeutics are summarized in the Table 1 below.

**Table 1:** Pharmacological therapies for sterile inflammation and their associated mechanisms.

Intervention	Mechanism	Species	Findings	Reference
Thrombomodulin	TLR-4/NF-κB/HMGB1 axis (inhibitor)	Mouse	Decreased expression of HMGB-1, downregulated caspase 3 expression and apoptosis, upregulated Bcl-2 (anti-inflammatory)	(168)
Dexmedetomidine	TLR-4/NF-κB/HMGB1 axis (inhibitor)	Rats	Decreased expression of cellular NF-κB, TLR-4, serum TNF-α, IL-1β, and myeloperoxidase	(169)
rhRelaxin	Apoptotic HMGB1 release (inhibitor)	Mouse	Reduced apoptosis frequency and caspase 3 expression, increased Bcl-xL/Bcl-2 expression, suppressed HMGB1 release	(24)
ML355	MAPK/NF-κB (inhibitor)	Mouse, porcine, non-human primate	Downregulation of proteins involved in MAPK pathway specific to the liver [12-hydroxyeicosatetraenoic acid (12-HETE)] and NF-κB	(170)
Dual specificity phosphatase 14 (Dusp14)	TAK1/Jnk1 TAK1/NF-κB (inhibitor)	Mouse	Decreased frequency of necrotic tissue, decreased neutrophil and macrophage infiltration, downregulation of caspase 3, downregulation of proinflammatory cytokines, upregulation of Bcl-2/Bcl-xL	(171)
mTOR (knockout model)	mTOR/NF-κB axis (prevents dysregulation)	Mouse	Decreased expression of caspase 3 and frequency of apoptosis, decreased expression of MCP1, TNFα, IL-6 (proinflammatory) cytokines	(172)
Omega-3 fatty acids	PI3k/Akt axis (activator)	Rat	Downregulation of NLRP3 and apoptosis, increased expression of glutathione, superoxide dismutase, and catalase (proteins that counteract oxidative stress), decreased expression of malondialdehyde, IL-18 and IL-1β (reflective of inflammation/oxidative stress)	(173)
Tanshinone IIa	PI3k/Akt axis (activator) TLR-4/NF-kB/HMGB1 axis (inhibitor)	Rat	Reduced expression of TNFα, IL-4, and HMGB1, lower frequency of cell death, increased expression of IL-10 and TGF-β (anti-inflammatory)	(172)
Regulator of G-protein signaling 14 (RGS14) (knockout model)	TAK1/Jnk1/p38 MAPK (inhibitor)	Mouse	Reduced frequency of cell necrosis, apoptosis (caspase 3 cleavage), and infiltrating immune cells, downregulation of Bcl-2 with upregulation of Bad and Bax, downregulation of inflammatory kines MCP1, TNFα, IL-1β	(174)
Serelaxin	Notch1 (activator)	Mouse	Reduced expression of MCP1, IL-1β, CXCL10, CXCL2 (inflammatory cytokines), markedly reduced immune cell infiltration, reduced frequency of apoptotic cells	(24)
Heme oxygenase 1 (knockout Model)	Heme oxygenase-1/sirtuin1/p53 axis	Mouse, human	Human: decreased caspase 3 cleavage (apoptotic frequency) Mouse: decreased macrophage activation, decreased MCP1, TNFα expression	(35)

### Machine perfusion

**Figure 3:**
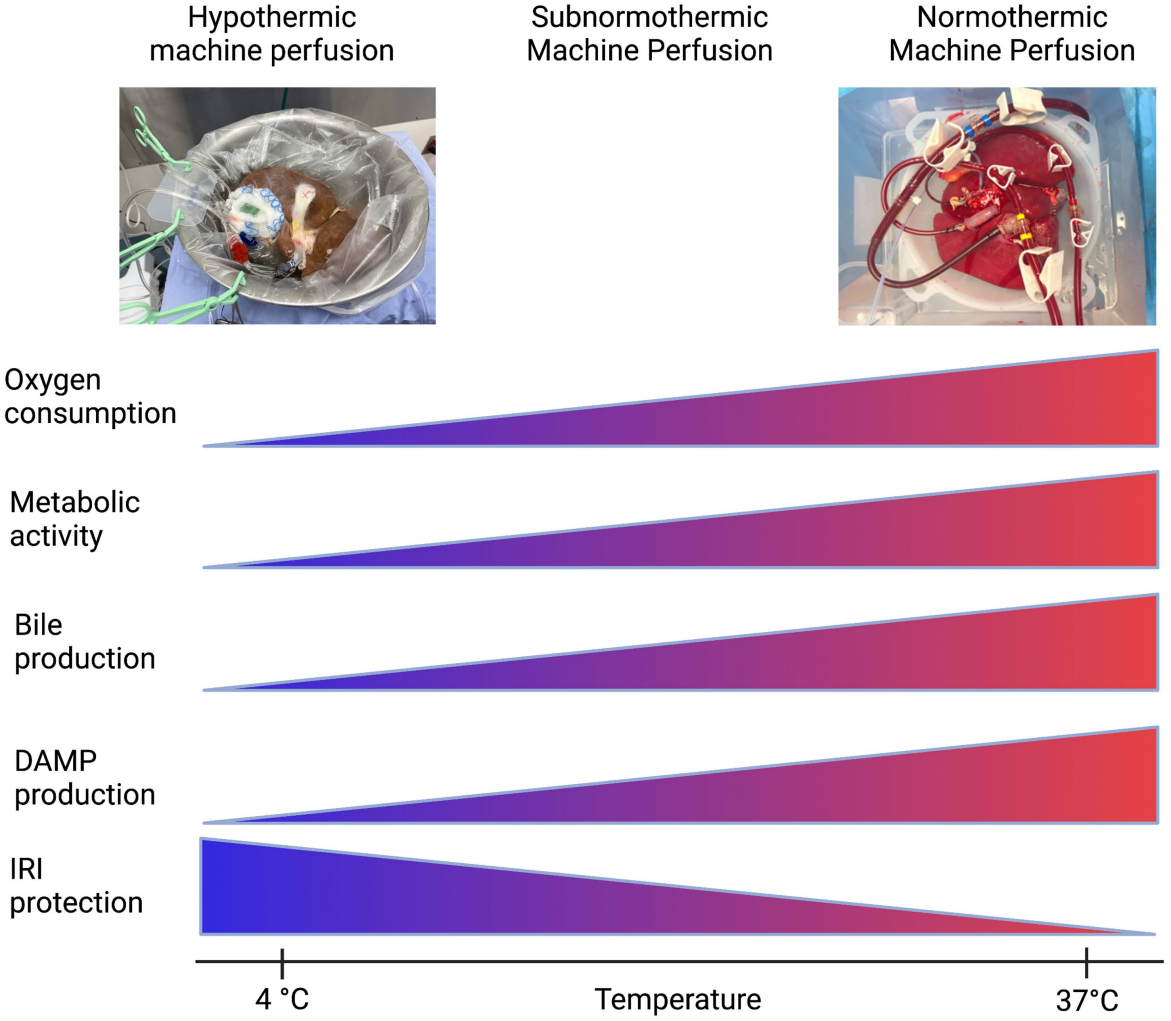
Machine perfusion: a balancing act. Created with biorender.com.

Preservation of the liver graft, a fundamental obstacle in liver transplantation, began with static cold storage (SCS), the first established preservation method for procured organs (175). Upon flushing the liver with cold preservation solution, cell metabolism, electron transport chain activity, and the production rate of succinate and reactive oxygen species (ROS) is significantly reduced in the tissue. Inevitably, however, cellular energy consumption continues ever so slowly, and with no oxygen present to oxidize end products of the electron transport chain, the tissue will still begin to accumulate harmful metabolic byproducts leading to ischemic injury (176, 177). Then, immediate exposure to normothermic temperatures and readily bioavailable oxygen sparks reperfusion injury; the return of abundant oxygen causes mass ROS to be produced and triggers sterile inflammation, which causes damage to tissue and further DAMP release (175). By employing machine perfusion as an intermediary, remedial preservation system, the prolonged cold storage period that sets the stage for this type of injury after organ transplantation could be eliminated. Machine perfusion has presented opportunity to improve outcomes in liver transplantation by reducing the length of prolonged ischemic time in donor organs and lessening tissue injury from DAMP release and sterile inflammation, as this is a major risk factor for the development of early allograft dysfunction (178, 179) (Figure 3). The mechanisms by which this type of injury occurs, and potential therapeutic targets, are more thoroughly reviewed in Dar et al. (180).

The first iteration of organ preservation by machine perfusion was in the form of non-oxygenated hypothermic machine perfusion (HMP). Even without supplemental oxygen, at HMP temperatures (4°C–8°C) the partial pressure of oxygen was hypothesized to be high enough in the circulating perfusate to satisfy the significantly reduced, but ever-present, oxygen demand of the slowly-metabolizing hepatic tissue. This modality, versus SCS, provided less potential for IRI and subsequent sterile inflammation upon implantation and reperfusion.

The next iterations of machine perfusion aimed to maintain slow tissue metabolism while allowing the organ access to increased levels of oxygen to more effectively prevent a backup in mitochondrial function and avoid severe IRI after implantation. These modalities were aptly named HOPE (hypothermic oxygenated perfusion) and DHOPE (dual oxygenation of the portal vein and hepatic artery during hypothermic perfusion) (181). van Rijn et al. (182) evaluated the efficacy of DHOPE in reducing bile duct injury after transplantation in a cohort of DCD livers, as biliary strictures due to epithelial damage are a frequent complication of liver transplantation. They found that DHOPE significantly reduced the severity of IRI in bile compartments of DCD livers after transplantation, and they hypothesized that the supplemental oxygen provided in their application of DHOPE, in tandem with the slowed metabolism of the mitochondria due to hypothermia, allowed for resuscitation of mitochondrial function, subsequent restoration of cellular ATP, and significant reduction of ROS production that would otherwise alter the structures of cell membranes and DNA molecules in a deleterious manner (182). In addition, the supplemental oxygen included in oxygenated hypothermic machine perfusion may have lessened the sterile inflammation response as it alone has been observed to lessen Kupffer cell activation and dampen HMGB1 release, attenuating activation of the innate and adaptive immune systems and resultant tissue damage (183).

Although HOPE and DHOPE proved to be promising preservation techniques compatible with successful liver transplantation and amelioration of post-implantation IRI, preservation under physiological conditions was still an appealing approach (30). Thus, oxygenated machine perfusion at normothermic (physiological) temperatures (NMP) was implemented (175). Nasralla et al. (184) executed a clinical randomized control trial to evaluate the efficacy of NMP vs. SCS; with increased supplemental oxygen to match the physiological mitochondrial metabolic rate at normothermic temperature, NMP displayed promise in improving outcomes post implantation. Jassem et al. (185) went further to uncover the mechanisms NMP may act on to ameliorate hepatic IRI after transplant. They found that in grafts subjected to NMP before transplant, release of proinflammatory cytokines and biomarkers were downregulated, including: IL-2, IL-6, IL-12, TNFα, and IFNγ. Additionally, sterile inflammation processes such as platelet activation, neutrophil infiltration, and activation of T lymphocytes were also downregulated (185). Recently, Clavien et al. (186) have successfully transplanted a human liver discarded by all transplant centers after 3 days of normothermic machine perfusion, and upon implant the graft displayed minimal signs of reperfusion injury, sterile inflammation, and need for immunosuppression.

Lastly, other groups such as (187, 188) have illustrated the efficacy of optimizing the balance between metabolic demand (controlled by temperature) and oxygenation level of the perfusate. In doing so, they have introduced a modality termed subnormothermic machine perfusion (SNMP): perfusion of an organ at room temperature (21°C), in an effort to keep the organ metabolizing at a level sufficient to allow assessment and treatment while keeping its demand for oxygen low enough to prevent IRI injury (187). This also allows for the use of an acellular perfusate, which is more readily available than blood based perfusates. Since the advent of DHOPE, the role of oxygen and the oxygen requirements of the organ at different temperatures suggests that a balance between protection from ischemia-reperfusion injury and the ability to assess the metabolites of the organ can be achieved.

The progression of machine perfusion is readily advancing from SCS with upper limits of 12 h to allowing for keeping an organ *ex vivo* for days and if progress continues, for weeks. Recovery of organs that were previously discarded such as in Clavien et al. (186), and maintaining viability for multiple days would greatly help with the graft shortage crisis and allow for an expansion of the distance that organs could reasonably travel to be given to a patient in need.

## Conclusion

Sterile inflammation in liver transplant still presents a clear and burdensome problem in ensuring allograft function and complication free survival. Recent advances into understanding the specific biomarkers of sterile inflammation may provide a real time ‘road map’ for understanding not only the grafts’, but also *the recipients*’ immune state and targeted therapeutics for reducing allograft dysfunction and long-term rejection. More exploration is needed to fully understand the implications of the recipient disease state and the particular challenges for allograft function.

## Author contributions

RK, PLC, NA, JP, and AB conceived this review. RK, PLC, and NA performed literature review and drafted the manuscript. RK, PLC, NA, and QG performed critical review and revision of the manuscript. MH, JP, and AB performed critical review and revision of the manuscript and oversaw the drafting process. All authors contributed to the article and approved the submitted version.

## Funding

This work was funded by NIH K08 AI150990 to AB and NIH R01 AI153274 to JP and AB.

## Conflict of interest

The authors declare that the research was conducted in the absence of any commercial or financial relationships that could be construed as a potential conflict of interest.

## Publisher’s note

All claims expressed in this article are solely those of the authors and do not necessarily represent those of their affiliated organizations, or those of the publisher, the editors and the reviewers. Any product that may be evaluated in this article, or claim that may be made by its manufacturer, is not guaranteed or endorsed by the publisher.
